# The PDZ Protein Canoe/AF-6 Links Ras-MAPK, Notch and Wingless/Wnt Signaling Pathways by Directly Interacting with Ras, Notch and Dishevelled

**DOI:** 10.1371/journal.pone.0000066

**Published:** 2006-12-20

**Authors:** Ana Carmena, Stephan Speicher, Mary Baylies

**Affiliations:** 1 Program in Developmental Biology. Sloan-Kettering Institute, Memorial Sloan-Kettering Cancer Center New York, New York, United States of America; 2 Instituto de Neurociencias de Alicante, Consejo Superior de Investigaciones Científicas/University Miguel Hernandez Unidad de Neurobiología del Desarrollo, Alicante, Spain; Cambridge University, United Kingdom

## Abstract

Over the past few years, it has become increasingly apparent that signal transduction pathways are not merely linear cascades; they are organized into complex signaling networks that require high levels of regulation to generate precise and unique cell responses. However, the underlying regulatory mechanisms by which signaling pathways cross-communicate remain poorly understood. Here we show that the Ras-binding protein Canoe (Cno)/AF-6, a PDZ protein normally associated with cellular junctions, is a key modulator of Wingless (Wg)/Wnt, Ras-Mitogen Activated Protein Kinase (MAPK) and Notch (N) signaling pathways cross-communication. Our data show a repressive effect of Cno/AF-6 on these three signaling pathways through physical interactions with Ras, N and the cytoplasmic protein Dishevelled (Dsh), a key Wg effector. We propose a model in which Cno, through those interactions, actively coordinates, at the membrane level, Ras-MAPK, N and Wg signaling pathways during progenitor specification.

## Introduction

In multicellular organisms, cells are exposed to a complex environment in which they read numerous and sometimes conflicting stimuli. Cross-communication between signaling pathways is crucial for the integration of the multiple intracellular responses elicited by simultaneous signals, allowing the generation of unique cell outputs. As a result of cross-communication, networks of signal interactions are established within the cell. The elucidation of the underlying mechanisms by which these networks are built and regulated is essential for the understanding and pharmacological treatment of pathologies in which signaling pathways are mis-regulated, such as some neural disorders and cancer [1].

The *Drosophila* mesoderm provides an excellent system for studying signaling networks as *Drosophila* can be subjected to complex genetic manipulations and multiple signaling pathways are coordinately involved throughout mesoderm differentiation. After gastrulation, uncommitted mesodermal cells migrate and proliferate. Then, autonomous and non-autonomous signals pattern the mesoderm, allocating regions from which progenitors of the different mesodermal tissues, such as the somatic muscles and heart, will arise [2]–[4]. Somatic muscle and heart progenitors are singled out from clusters of equivalent cells (“promuscle groups”) that express the transcription factor Lethal of scute (L'sc), in a process reminiscent of neural progenitor specification [5]. These progenitors divide asymmetrically to give rise to two founder cells [6], [7]. Each founder cell is endowed with a unique identity by expressing specific combinations of transcription regulators such as Slouch/S59, Krüppel (Kr) or Even-Skipped (Eve) [4], [8]. We have focused on the specification of a dorsal subset of muscle and heart progenitors that express the identity protein Eve [9]. These progenitors differentiate upon the concerted and combinatorial action of four highly conserved signal transduction pathways triggered by Wingless (Wg)/Wnt, Decapentaplegic (Dpp)/TGF-*β*, Receptor Tyrosine Kinase (RTK)-Ras-MAPK and Notch (N) [10]. Cross-talk between Ras and N signaling pathways throughout Eve^+^ progenitor specification has been previously reported [11]. Indeed, crosstalk between N and Ras signaling pathways is necessary for signal integration in multiple processes during *Drosophila* development, as well as in other invertebrate and vertebrate systems [12]–[18]. However, the mechanisms by which N and Ras pathways cross-communicate are only beginning to be elucidated [19]–[23].

In this work, we have investigated the function of the *Drosophila* PDZ (PSD-95, Dlg, ZO-1) domain-containing protein Canoe (Cno) as a regulator of N and Ras cross-communication throughout Eve^+^ progenitor specification. *cno* mutant alleles were originally isolated by their dorsal open phenotype, hence its name (Jürgens et al, 1984). Indeed, Cno has been shown to participate in the morphogenetic process known as dorsal closure of the epidermis by modulating the Jun N-terminal kinase (JNK) signaling cascade (Takahashi et al.1998). *cno* encodes a cytoplasmic protein associated with cell junctions in epithelial tissues, where Cno function has been studied in *Drosophila* [24], [25]. The human ortholog of Cno, AF-6, was initially identified as a fusion partner of ALL-1, a product involved in human leukemias [26]. AF-6 also localizes at epithelial cell junctions where it binds ZO-1, a tight-junction protein important for cell-cell contacts and cytoskeleton rearrangement [27], [28]. Cno and AF-6 share a similar structure: one PDZ motif, characteristic of scaffolding proteins associated to the plasma membrane that contribute to the subcellular spatial organization of signaling pathways [29]–[31]; a Kinesin-like domain and a Myosin-V-like domain, characteristic motifs present in proteins that interact with cytoskeleton components [32]; and two Ras-associating motifs [33] through which Cno/AF-6 binds to the activated form of Ras (Ras^Act^) [34]. In *Drosophila*, Cno interacts genetically with Ras, during eye development, and with *N,* during bristle and wing development [24], [35], [36]. However, the involvement of Cno in N-Ras cross-talk has not been studied. Here we propose a model in which Cno mediates N-Ras cross-talk through Dishevelled (Dsh/Dvl), the most proximal cytoplasmic component of Wg/Wnt pathway [37]–[40]. We suggest that Cno, by binding Ras^Act^, Dsh and N, represses the signals that these proteins trigger and actively coordinates, at the membrane level, RTK-Ras-MAPK, Dl-N and Wg-Dsh signaling pathways throughout progenitor specification.

## Results

**Figure 1:**
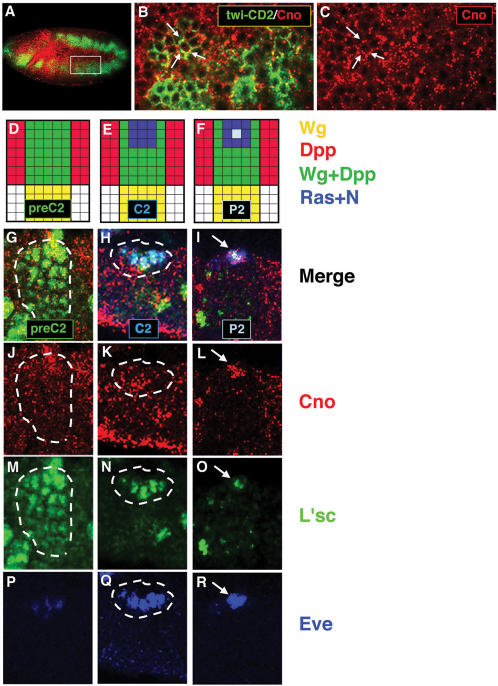
Cno is expressed in the mesoderm throughout progenitor specification. (A) Confocal immunofluorescence showing a lateral view of a late stage 10 embryo. Cno (red) is detected in the mesoderm (green). (B, C) Higher magnification of two hemisegments (63×; inset in A) reveals punctuate Cno expression at submembrane locations (arrows). (D–F) Diagrams show the most dorsal part of one hemisegment and the signals involved throughout dorsal progenitor specification. (G–R) Confocal immunofluorescences showing high magnification (63×) of the most dorsal part of one hemisegment. (D, G, J, M) Cno is expressed in the dorsal mesodermal region pre-patterned by Wg and Dpp (preC2) along with L'sc. (E, H, K, N, Q) Cno is detected in the equivalence group in which Ras is locally activated (C2) restricting L'sc to this cluster and activating Eve. (F, I, L, O, R) Cno is expressed in the progenitor (P2) singled out from this cluster after N-promoted lateral inhibition.

The specification of dorsal somatic muscle/heart progenitors depends on the combinatorial action of multiple signaling pathways [10]. First, Wg/Wnt and Dpp/TGF-β, secreted from the ectoderm to the mesoderm, define a dorsal region (preC2) revealed by the expression of the transcription factor L'sc. Then, two RTKs, the *Drosophila* EGF receptor (Egfr) [41] and the FGF receptor Heartless (Htl) [42] trigger a positive Ras signal in two groups of equivalent cells, within the dorsal mesodermal region prepatterned by Wg and Dpp (cluster C2, shown in Fig. 1, and cluster C15, not shown). RTK-Ras, through the MAPK module, upregulates L'sc expression and activates the identity protein Eve in these clusters, which appear sequentially. In addition, Delta (Dl) [43] activates its receptor N, promoting a lateral inhibitory signal among the cells of the equivalence group, so that only one progenitor is singled out from each cluster (progenitor P2, Fig. 1D–1F; and progenitor P15, not shown).

**Figure 2:**
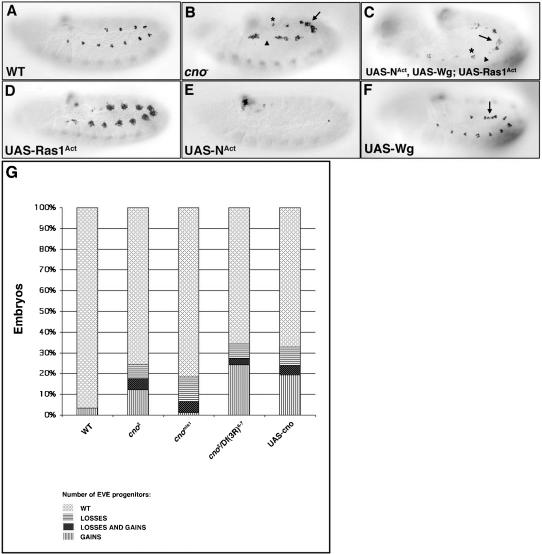
*cno^2^* zygotic null mutants display gain and loss of progenitors. All panels show lateral views of stage 11 embryos stained with an α-Eve antibody. (A) Eve wildtype (wt) expression in a subset of dorsal muscle/heart progenitors and founder cells (2–3 cells) per hemisegment. (B) *cno^2^* mutants show gain (arrow), loss (arrowhead) or a wildtype number (asterisk) of Eve^+^ progenitors in different hemisegments. (C) Embryos in which N^Act^, Wg and Ras^Act^ are simultaneously expressed in the mesoderm display a *cno^2^*-like phenotype: gain (arrow), loss (arrowhead) and wildtype number of Eve^+^ progenitors (asterisk). (D) Overexpression of Ras1^Act^ in the mesoderm induces the specification of extra Eve^+^ progenitors. (E) Overexpression of N^Act^ in the mesoderm inhibits Eve^+^ progenitor specification. (F) Embryos in which Wg is overexpressed in the mesoderm show gain of Eve^+^ progenitors in intersegmental regions (arrow). (G) Table shows % of embryos of the indicated genotype that display a wildtype number of Eve^+^ progenitors, Eve^+^ progenitors gain, loss and both (gain and loss). *cno^2^* and *cno^mis1^* are null and hypomorph mutant alleles, respectively; *Df(3R)^6-7^* removes *cno*. *cno* gof phenotype (*UAS-cno*) is also variable. Legends for bars are shown below the table. At least 70 embryos of each genotype were counted. P<0.0001 for all genotypes except for *cno^mis1^* (P = 0.0017).

Loss-of-function (lof) analysis of Ras-MAPK signaling pathway components reveals a decrease in the number of progenitors specified. Conversely, lof mutants of components of N signaling pathway display an increased number of progenitors. The gain-of-function (gof) phenotypes have the opposite effect: overexpression of Ras^Act^ in the mesoderm leads to the specification of extra progenitors whereas overexpression of an activated form of N (N^Act^) inhibits progenitor specification [11] (see also Fig. 2D and 2E). These effects are tightly linked, as Ras-MAPK and N signaling pathways act in concert. A complex network of competitive and cooperative interactions between Ras and N signal transduction pathways during muscle/heart progenitor specification has been documented. For example, N signaling downregulates the expression of different components of Ras-MAPK pathway and Ras signaling upregulates the N ligand Dl [11]. Hence, an accurate regulation of N and Ras signaling thresholds is critical for the acquisition of a progenitor fate. Since Cno has been shown to genetically interact with N or Ras signaling in different tissues and to physically interact with Ras^Act^ (see introduction), we analyzed a possible function of Cno during progenitor formation modulating N-Ras cross-talk.

### Cno is expressed in the Drosophila embryonic mesoderm throughout muscle/heart progenitor specification

As the first step, we asked whether Cno is expressed in the mesoderm during progenitor specification. Cno expression was examined in embryos that carry a *twi-CD2* insertion, which drives the expression of the transmembrane protein CD2 under the control of a mesoderm-specific *twist* (*twi*) promoter. We observed that Cno was expressed in the mesoderm in a punctuate pattern close to the cell membrane (Fig. 1A–1C).

To investigate whether Cno was expressed when N and Ras signaling pathways interact to specify dorsal progenitors, we analyzed Cno expression with different progenitor markers. Cno was detected along with L'sc, the earliest and most general progenitor marker, in the dorsal region pre-patterned by Wg and Dpp (preC2). Cno was also detected along with the identity protein Eve in the dorsal Eve^+^ equivalence group (C2) and, subsequently, in the progenitor (P2) (Fig. 1D–1R). Thus, Cno was expressed in the mesoderm during the time when Ras and N are regulating the specification of dorsal progenitors.

### cno zygotic mutant embryos show gain and loss of Eve progenitors

To investigate a potential function of Cno in Ras-N crosstalk during progenitor specification, we analyzed the dorsal Eve^+^ progenitors in *cno* ^2^ zygotic null mutant embryos. Three different phenotypes were observed: (1) wildtype-like hemisegments, (2) hemisegments with extra Eve^+^ progenitors and (3) hemisegments displaying loss of Eve^+^ progenitors. Comparable results were obtained with different *cno* mutant alleles (Fig. 2B and 2G). Likewise, the expression of other muscle progenitor markers and signaling proteins involved in muscle/heart progenitor specification were, like Eve, both upregulated or downregulated in different hemisegments of *cno^2^* zygotic null mutant embryos (Fig. S1A–S1R). These effects on muscle progenitor specification were reflected in the final muscle pattern where gain and loss of specific body muscles was observed (Fig. S1S–S1V). The progenitor markers (including Eve) and signaling proteins analyzed (Fig. S1) are under Ras and N regulation [11]. Indeed, the *cno* ^2^ mutant phenotype resembled a mosaic of Ras and N mutant phenotypes (Fig. 2A, 2B, 2D and 2E) [11]. Given the reported relationship between Cno and Ras or N signaling pathways in different systems (see introduction), we hypothesized that Cno could be modulating both Ras and N signaling during muscle progenitor formation. Thus, we examined whether Cno interacted with these signaling pathways during progenitor specification.

### cno genetically interacts with N and Ras signaling pathways during progenitor specification

**Figure 3:**
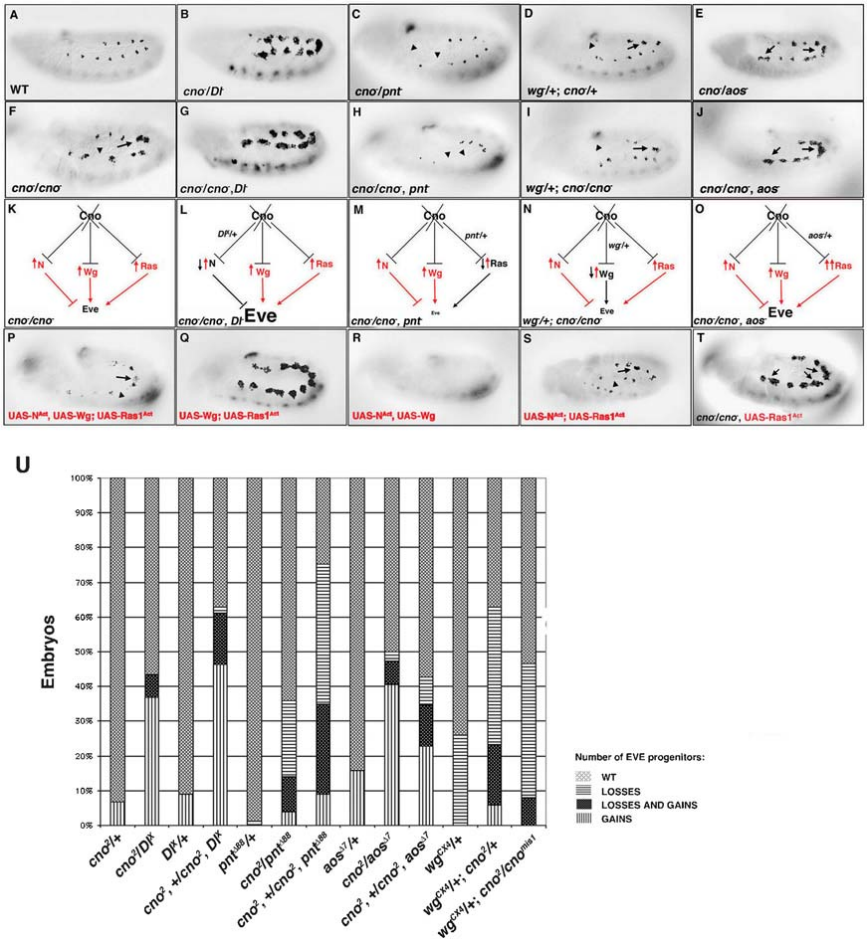
*cno* interacts genetically with N, Ras and Wg signaling pathways. (A–J and P–T) Lateral views of stage 11 embryos stained with an *α*-Eve antibody. (B, G) Extra Eve^+^ progenitors are detected both in *cno^2^/Dl^X^* transheterozygotes and in *cno^2^/cno^2^*, *Dl^X^* embryos. (C, H) Both *cno^2^/pnt^*Δ*88^* double heterozygotes and *cno^2^, pnt^*Δ*88^/cno^2^* embryos show loss of Eve^+^ progenitors. (E, J) An increase in Eve^+^ progenitors is observed both in *cno^2^*/*aos^*Δ*7^* transheterozygotes and in *cno^2^*, *aos^*Δ*7^*/*cno^2^* embryos [74]. (D, I) Gain and loss of Eve^+^ progenitors are detected both in *wg^CX4^/+*; *cno^2^/+* double heterozygotes and in *wg^CX4^/+*; *cno^mis1^/cno^2^* embryos. (F–I, K–N, P–S): (P) Simultaneous mesodermal overexpression of N^Act^-Wg-Ras^Act^, (Q) Wg-Ras^Act^, (R) N^Act^-Wg and (S) N^Act^-Ras^Act^ have similar phenotypes than *cno^2^/cno^2^* (F, K), *cno^2^/cno^2^*, *Dl^X^* (G, L), *cno^2^/cno^2^*, *pnt^*Δ*88^* (H, M) and *wg^CX4^/+*; *cno^mis1^/cno^2^* (I, N) embryos, respectively. Arrows and arrowheads indicate gain and loss of progenitors, respectively. (K–O) Diagrammatic representation of the effect on Eve expression of *cno* lof (K) and *cno* lof in combination with reduced doses of N (L), Ras (M) and Wg (N) signaling components, or with increased doses of Ras signal (O). (T) The phenotype of Ras1^Act^ mesodermal overexpression is enhanced in a *cno^2^* mutant background. (U) Table shows % of embryos of the indicated genotype that display a wildtype number of Eve^+^ progenitors, Eve^+^ progenitors gain, loss and both (gain and loss). Legends for bars are shown right to the table. At least 70 embryos of each genotype were counted. P<0.0001 for all genotypes except for *cno^2^/cno^2^*, *aos^*Δ*7^* (P = 0.0239) and *wg^CX4^*/+; *cno^2^*/*cno^mis1^* (P = 0.006).

A classical genetic approach to uncover gene interactions during biological processes is the analysis of transheterozygous mutant phenotypes. Thus, to determine if Cno was interacting with N signaling during progenitor specification, we examined Eve expression in embryos with reduced doses of *cno* and different components of the N signaling pathway, including Dl, the ligand of N required for progenitor specification, and N itself. For example, double heterozygotes of *cno* and *Dl* (*cno^2^*/*Dl^X^*) showed a great expansion of Eve^+^ progenitors, which was not observed in the single heterozygotes *cno^2^*/+ and *Dl^X^/+* (Fig. 3B and 3U). Further genetic interactions (Fig. 3G, 3U and not shown) indicated that *cno* interacts with N signaling during muscle progenitor formation.

In a similar way, transheterozygous mutant analysis revealed strong genetic interactions between *cno* and Ras pathway components, including *pointed* (*pnt*), which encodes an ETS-domain effector of Ras signaling [44] and Ras itself (Fig. 3 and not shown). For example, double heterozygotes for *cno* and *pnt* (*cno^2^*/*pnt^*Δ*88^*) showed a great loss of Eve^+^ progenitors, which was not observed in the single heterozygotes *cno^2^/+* and *pnt^*Δ*88^/+* (Fig. 3C and 3U). Additional genetic interactions (Fig. 3H, 3E, 3J, 3U and not shown) indicated that Cno functions with Ras signaling during progenitor specification. In summary, our data revealed genetic interactions between Cno and both N and Ras-MAPK signaling pathways.

### cno interacts genetically with Wg signaling pathway

**Figure 4:**
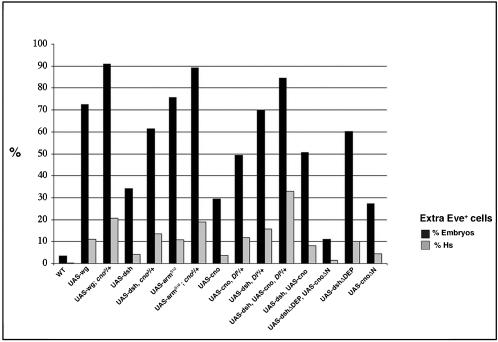
Cno inhibits Wg-Dsh pathway and links it with Dl-N pathway. Table shows % of embryos and hemisegments (Hs) for each indicated genotype that display extra Eve^+^ cells. At least 70 embryos/770 hemisegments were counted in each case. P<0.001 for all genotypes except for UAS-wg; *cno^2^*/+, “% Embryos bar” (P = 0.0027); UAS-arm^S10^, *cno^2^*/+, “% Embryos bar” (P = 0.0337); UAS-cno, *Dl^x^*/+, “% Embryos bar” (P = 0.0177); UAS-dsh, UAS-cno, *Dl^x^*/+, “% Embryos bar” (P = 0.0472); UAS-dsh, UAS-cno, “% Embryos bar” (P = 0.0115), “% Hs bar” (P = 0.0002).

The canonical Wg signaling pathway is another critical component for dorsal muscle/heart progenitor specification. *wg* lof mutants show a loss of Eve^+^ progenitors, whereas Wg overexpression in the mesoderm induces extra Eve^+^ progenitors; these additional progenitors frequently appear in intersegmental regions [10], [45] (Fig. 2F). Intriguingly, the extra Eve^+^ progenitors specified in *cno^2^* null mutant embryos were often observed in intersegmental regions (Fig. 2B). Furthermore, Cno has been shown to interact genetically with some components of the Wg pathway during wing morphogenesis [24], [46]. Hence, we investigated whether *cno* genetically interacted with components of the Wg pathway during progenitor specification. Transheterozygotes for *wg* and *cno* (*wg^CX4^*/+; *cno^2^*/+) revealed a strong interaction (Fig. 3D and 3U). Additional genetic interactions were found between *cno* and components of the canonical Wg pathway that further supported a functional relationship between Cno and Wg pathway during progenitor specification (Fig. 3I, 3U and Fig. 4, see below).

### Cno inhibits N, Ras and Wg signaling pathways

Our genetic data indicated that Cno interacts with Wg, Ras and N signaling pathways throughout the process of dorsal muscle progenitor specification. These three signaling pathways act as positive (Wg and Ras) and negative (N) signals during progenitor formation [10]. We hypothesized that Cno regulates the relative thresholds of those positive and negative signals throughout progenitor specification. To further understand how Cno affects Wg, Ras and N signaling pathways during this process, we analyzed in more detail the functional relationships between Cno and each of these pathways.

Several experiments revealed an inhibitory effect of Cno on N signaling. For example, the *cno* overexpression phenotype was enhanced when N activity was reduced (*Dl^X^* heterozygous background) (Fig. 4). In addition, the N effector and transcriptional repressor, E(spl)-m8, was upregulated in *cno* ^2^ null mutants (Fig. S2C and S2D). Thus, Cno showed an antagonist effect on N signaling.

Additional genetic interactions between Cno and the RTK-Ras-MAPK pathway also suggested an inhibitory effect of Cno on Ras-MAPK signaling during progenitor specification. For example, the Ras^Act^ overexpression phenotype was enhanced in a *cno^2^* homozygous background (Fig. 3T). In addition, examples of synergism between Cno and negative regulators of Ras signaling were found (Fig. 3E, 3J and Fig. S3). These genetic data supported a repressive effect of Cno on Ras-MAPK signaling.

Lastly, our experiments suggested that Cno, under wildtype conditions, also antagonized canonical Wg signaling. For example, we found that *cno* lof dominantly enhanced the overexpression phenotype of Wg, Dsh and Armadillo (Arm)/β-catenin [47] (Fig. 4). In addition, cuticles from *cno^2^* mutant embryos showed a naked phenotype, characteristic of Wg overexpression (not shown). These genetic interactions, in addition to the *cno^2^* null mutant phenotype, suggested that Cno has an inhibitory effect on canonical Wg signaling.

Altogether, our genetic data supported an inhibitory effect of Cno on N, Ras-MAPK and Wg signaling pathways. We thus expected an increase in activity of all these signaling pathways in *cno^2^* mutants. Indeed, the simultaneous overexpression of Wg, N^Act^ and Ras^Act^ in the mesoderm phenocopied the *cno^2^* null mutant phenotype (Fig. 3F, 3K, and 3P). Furthermore, the phenotypes observed by reducing N, Ras or Wg signaling in* cno^2^* mutants resembled those found by simultaneously overexpressing Wg-Ras1^Act^, N^Act^-Wg or N^Act^-Ras^Act^, respectively (compare Fig. 3G, 3L, 3Q; 3H, 3M, 3R and 3I, 3N, 3S). The inhibitory effect of Cno on N, Ras and Wg signaling pathways may seem in conflict with the phenotypes observed in some of the genetic combinations for *cno* and components of the N, Ras or Wg pathways (Fig. 3). However, the upregulation of both positive and negative signals in *cno^2^* mutant embryos can explain the net effect on Eve expression found in the different genetic combinations. For example, in *cno^2^/cno^2^, pnt^*Δ*88^* embryos (Fig. 3H and 3M), there is an increase in all three signals: the two positive (Ras, Wg) and one negative N signal. However, the Ras pathway is impaired in these embryos (*pnt^*Δ*88^*/+ genetic background). In addition, N signaling, which downregulates the Ras pathway [11], increases in *cno^2^/cno^2^, pnt^*Δ*88^* embryos. Hence, the net effect is a great reduction of the positive Ras signal and thus, a great reduction of Eve expression.

### Cno physically interacts with N and Dsh

**Figure 5:**
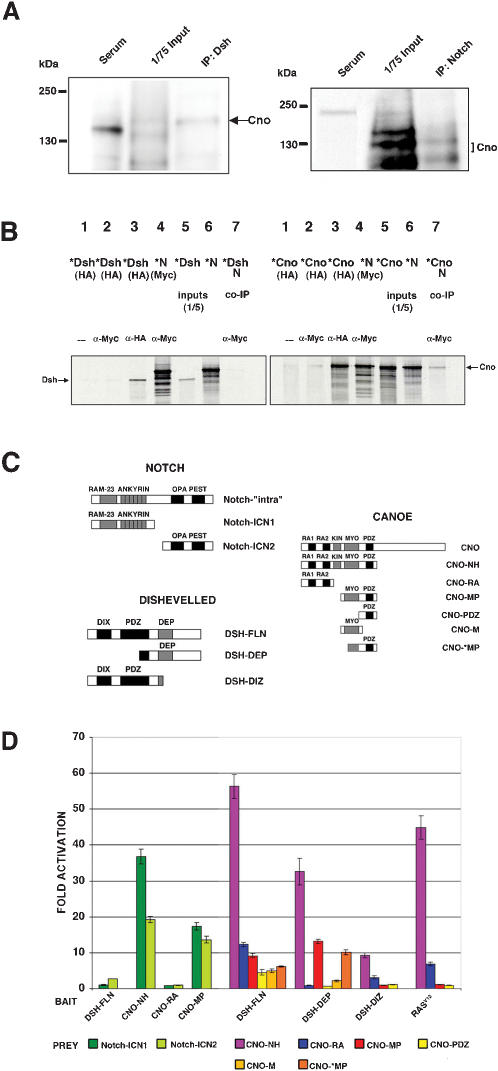
Cno physically interacts with Dsh and N. (A) Coimmunoprecipitations from embryonic lysates. Left panel: Cno is coimmunoprecipitated with endogenous Dsh. Right panel: Cno is coimmunoprecipitated with endogenous N. Different sizes of the Cno protein coimmunoprecipitated with Dsh and N (see text). A Rat and Rb sera without the Dsh or N antibodies were used as negative controls. (B) In vitro coimmunoprecipitations (see also Fig. 7I). Left panel: HA-tagged Dsh (DSH-FLN) does not coimmunoprecipitate with N. Right panel: HA-tagged Cno (CNO-NH) coimmunoprecipitates with Myc-tagged N (Notch-“intra”). In each panel, lanes 1 and 2: ^35^S-labelled (*) protein, HA-tagged, without adding any antibody (Ab) (lane 1) or adding α-Myc Ab (lane 2), as negative controls; lanes 3 and 4: *proteins HA-tagged (lane 3) or Myc-tagged (lane 4) tested with the corresponding Ab; lanes 5 and 6: translation products; lane 7: coimmunoprecipitation of the *HA-tagged protein with the Myc-tagged protein by an *α*-Myc-Ab. (C) N intracellular domain (Notch-“intra”), Dsh and Cno constructs used in the yeast two-hybrid and co-IP assays (see also “Materials and methods”). (D) Yeast two-hybrid: averaged results of at least four independent protein extracts in *β*-galactosidase quantitative liquid assays. The fold activation determined with respect to the corresponding empty vector is represented. Bars indicate s.e.m. DSH-FLN, CNO-NH, CNO-RA and CNO-MP were tested against Notch constructs: Notch-ICN1 and Notch-ICN2. DSH-FLN, DSH-DEP, DSH-DIZ and RAS^V12^ were tested against different Cno constructs (C).

Wg, Ras and N signaling pathways are critical for muscle progenitor specification: Wg signaling first sets up a region of competence, in which Ras and N will subsequently be locally activated to specify a single progenitor (Fig. 1). Our mutant analysis and genetic interaction data suggested that Cno antagonizes all three pathways, Ras-MAPK, N and Wg, during progenitor specification. The reported direct binding of Cno/AF-6 to Ras^Act^ [34] could explain the Cno repressive effect on Ras-MAPK signaling. Indeed, AF-6 competes with Raf-1, a main Ras effector, for binding Ras [34]. We confirmed this physical interaction between Cno and Ras^Act^ (Fig. 5D). To understand how Cno inhibited N and Wg signaling, we examined potential physical interactions between Cno and members of the N and Wg signaling pathways. Both N and Dsh, a key Wg effector, have the consensus binding site for the PDZ domain of Cno/AF-6 at their C-terminus [48]. Hence, we analyzed physical interactions between Cno, N and Dsh.

**Figure 6:**
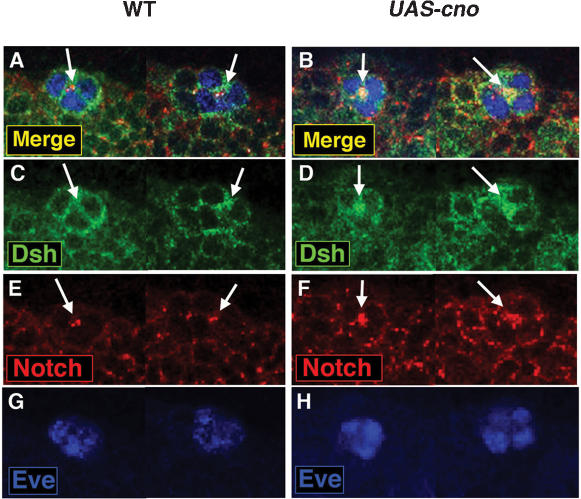
Overexpression of Cno leads to the stabilization of Dsh and N close to the membranes of Eve^+^ interacting cells. The most dorsal part of two hemisegments of stage 11 embryos are shown in all panels (63× magnification). Eve marks a dorsal equivalence group in the mesoderm (blue). (A, C, E, G) In wildtype (WT) embryos, Dsh (green) and N (red) colocalize at some regions in Eve^+^ cell clusters (arrows). (B, D, F, H) Cno overexpression (*UAS-cno*) leads to an increase of Dsh and N close to the membranes of Eve^+^ cells (arrows).

**Figure 7:**
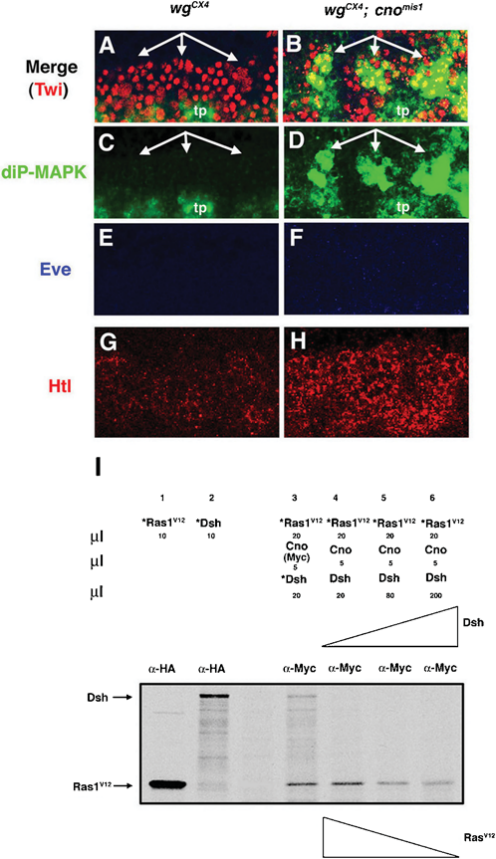
Wg-Dsh signaling releases Cno-mediated Ras repression. (A–H) Lateral views at high magnification (63×) of stage 11 embryos showing three hemisegments. (A, C, E) In *wg^CX4^* mutants neither diP-MAPK (green) nor Eve (blue) expression is detected in the mesoderm, which is marked by Twi (red). (G) The FGFR Htl (red) is also downregulated in *wg^CX4^* mutants. (B, D, F, H) Both diP-MAPK (B, D) and Htl (H) expression are rescued in *wg^CX4^; cno^mis1^* double mutants; Eve expression is not rescued (F). tp, tracheal pits. (I) Dsh competes with Ras for binding Cno. Proteins were translated in vitro in presence (*) or absence of ^35^S-Methionine. Lane 1 and 2: *Ras^V12^ and *Dsh, both HA-tagged, were immunoprecipitated with an *α*-HA antibody (Ab). Lane 3: Both *Ras^V12^ and *Dsh were co-IP with Cno by an *α*-Myc Ab. Lane 4, 5 and 6: The addition of increasing amounts of Dsh leads to a decrease in the amount of *Ras^V12^ co-IP with Cno.

We carried out coimmunoprecipitations (co-IPs) using both embryonic lysates and in vitro translated proteins. These experiments showed that Cno coimmunoprecipitates with both N and Dsh (Fig. 5A, 5B and Fig. 7I). The co-IPs from embryonic lysates showed different sizes of the Cno proteins coimmunoprecipitated with either Dsh or N (Fig. 5A). We suggest that some of these different forms of Cno are due to different post-translational modifications in each of those complexes, different isoforms of Cno or/and specific breakdown products of Cno (See also Fig. S5). To characterize the domains implicated in the binding, diverse deletion constructs of Cno, N and Dsh were tested in yeast two-hybrid assays (Fig. 5C and 5D). Only Cno constructs comprising the PDZ domain strongly interacted with both proximal (Notch-ICN1) and distal (Notch-ICN2) regions of the N intracellular domain (approximately 40 and 20 fold activation, respectively). The Cno-Dsh interaction, however, mapped to two different domains of Cno: the PDZ domain and the Ras-binding domain (Fig. 5C and 5D). To distinguish which domains in Dsh bound the Ras-associating and PDZ motifs of Cno, different Dsh constructs were tested. A Dsh fragment containing the DIX (DIshevelled, aXin) and PDZ domains (DSH-DIZ) only bound Cno constructs containing the Ras-binding domain, as did the Ras positive control (Fig. 5C and 5D). In contrast, the Dsh fragment containing the DEP (Dsh, egl-10, pleckstrin) domain (DSH-DEP) only bound Cno constructs containing the PDZ domain plus some of the adjacent Myosin-V (Myo)-like domain (Fig. 5C and 5D). Thus, these data indicated that Cno physically interacted with both N and Dsh. These physical interactions could lead to the inhibitory effects of Cno on N and Wg-Dsh signaling pathways.

### Cno links Wg-Dsh and Dl-N signaling pathways

Dsh has been shown to interact physically with N and to inhibit its signaling during *Drosophila* wing bristle development [49]. We found that, genetically, Dsh also acted antagonistically to N signaling during muscle/heart progenitor specification (Fig. S2A, S2B and Fig. 4). For example, the phenotype of Dsh mesodermal overexpression was enhanced by reducing N signaling activity (*Dl^X^*/+ genetic background) (Fig. 4). However, the mechanism underlying the Dsh-N antagonism is unclear: the reported Dsh-N (Notch-ICN2) physical interaction was very weak under our conditions (approximately 3 fold activation, Fig. 5D; see also Discussion) [49], [50]. Moreover, Dsh failed to co-immunoprecipitate in vitro with N under our conditions (Fig. 5B). Hence, although Dsh had an antagonistic effect on N signaling during muscle progenitor specification, our data did not support a strong physical interaction between Dsh and N as the means by which Dsh inhibits N signaling. Dsh has been shown to co-immunoprecipitate with N in vivo [51]. This interesting finding supports that Dsh and N are forming part of a complex during embryogenesis. However, it is not a proof of a direct physical interaction between both proteins. Since (1) Cno had a negative effect on N signaling, (2) Cno coimmunoprecipated with N in vivo, (3) Cno and N proteins directly interacted in vitro and in yeast two-hybrid assays and (4) Cno interacted physically with Dsh, we reasoned that Cno could mediate the repressive effect of Dsh on N signaling.

To test this function of Cno as a mediator of the inhibitory effect of Dsh on N, we performed several genetic experiments. For example, the phenotype of Dsh overexpression under conditions of reduced N activity (*Dl^X^*/+ genetic background, see above) was further enhanced by the simultaneous overexpression of Cno (Fig. 4). This result supported a function of Cno in mediating the N repression by Dsh. The relevance of Cno-Dsh interaction to inhibit N signaling was further supported by using specific deletion constructs of Dsh (Dsh*Δ*DEP) and Cno (Cno*Δ*N) that cannot interact with each other. Whereas the simultaneous mesodermal overexpression of wildtype forms of Dsh and Cno led to a great increase of Eve^+^ cells (a N lof phenotype), the simultaneous overexpression of Dsh*Δ*DEP (lacks the DEP domain) [52] and Cno*Δ*N (lacks the RA1 and RA2 domains) [53] (see also Fig. 5) showed a much weaker effect, consistent with the existence of more N activity in the mesoderm of these embryos (Fig. 4; compare with overexpression of wildtype forms of Dsh and Cno).

The DEP domain of Dsh is required to relocate Dsh at the membrane [54]. Given the physical interaction between Cno and the region of Dsh that contains the DEP domain, we next examined whether Cno was affecting Dsh cell localization. Cno overexpression in the mesoderm led to a striking accumulation of both Dsh and N close to the membrane of Eve^+^ interacting cells (arrows in Fig. 6B, 6D and 6F; compare to 6A, 6C, and 6E). This observation suggested that the negative effect of Cno on N and canonical Wg pathway could be related to the accumulation of Dsh and N at the cell membrane (see Discussion). Taken together with the genetic and physical interaction data, these results supported a cross-regulatory interaction between N and Dsh mediated by Cno during progenitor formation.

### Cno links Wg-Dsh and Ras-MAPK signaling pathways

Wg signaling provides mesodermal cells competence to respond to Ras signaling, in part through the requirement of Wg to maintain the expression of different components of the Ras pathway [55]. For example, in *wg* mutant embryos, we detected reduced expression of the *Drosophila* FGF receptor Htl, as well as no expression of diP-MAPK, a measure of Ras activation (Fig. 7C and 7G; [56]). However, the molecular mechanism by which this competence is achieved, either by direct regulation or indirectly through unknown mediators, is not completely understood. Since Cno bound and inhibited Ras, and the Wg effector Dsh bound the Cno Ras-associating domain, we hypothesized that Wg signaling would release Cno-mediated Ras inhibition through competition between Dsh and Ras for the Cno Ras-binding domain. Therefore, we predicted that some Ras activity would be rescued in *wg* mutants in which Cno is no longer present to repress Ras. To test this hypothesis, we analyzed MAPK activity and the expression of proteins under Ras regulation in *wg^CX4^*; *cno^mis1^* double mutants. DiP-MAPK expression, which is lost in *wg^CX4^* mutants (Fig. 7A and 7C, compare to Figure S3A and S3B) [55], was partially rescued in *wg^CX4^; cno^mis1^* double mutants (Figure 7B and 7D). This was the same level of rescue obtained in *wg^CX4^* mutant embryos by overexpression of Ras^Act^ [55]. As expected, Eve expression was not rescued in *wg^CX4^; cno^mis1^* mutant embryos (Fig. 7A, 7B, 7E and 7F) nor is it rescued in *wg^CX4^* mutant embryos by Ras^Act^, as Eve expression, in addition to Ras, requires a direct transcriptional input from Wg signaling [10], [55], [57]. Moreover, the expression of the *Drosophila* FGF receptor Htl [58], which is downregulated in *wg^CX4^* mutant embryos, was also rescued in *wg^CX4^; cno^mis1^* double mutants (Fig. 7G and 7H) [55]. Since Ras-MAPK upregulates Htl expression [11], the increase of Htl expression in *wg^CX4^; cno^mis1^* double mutants was consistent with the rescue of MAPK activity found in these embryos. These results suggested that Wg signaling releases the Ras repression mediated by Cno.

**Figure 8:**
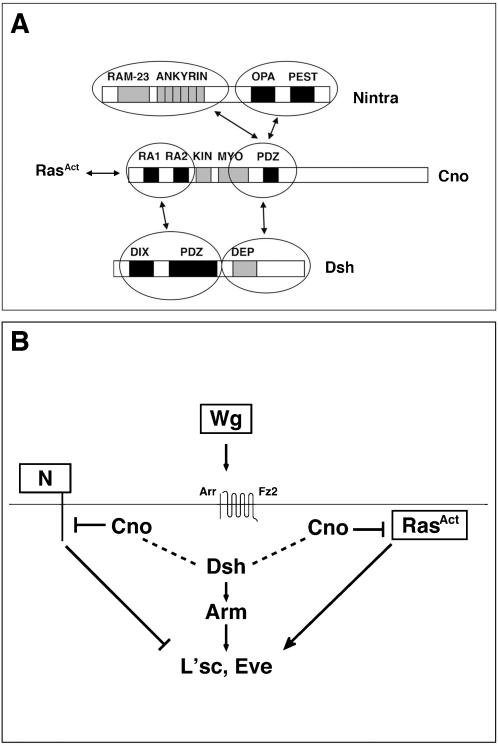
Summary of physical interactions and working model. (A) Diagram summarizing the physical interactions between Cno and Nintra, Ras^Act^ and Dsh. (B) Working model. The modulation of the information conveyed by three signaling pathways, Wg, N and Ras-MAPK, is critical for muscle progenitor specification. Our data show that Cno interacts physically and genetically with these three pathways, negatively regulating the information flow to key progenitor transcriptional targets, L'sc and Eve. In this model, the Cno/Dsh interaction is pivotal to modulate signaling thresholds. Cno/Dsh interaction facilitates the release of Cno repression on Ras^Act^ signaling and the inhibition of N signaling by Cno. Dsh recruitment by Cno leads to canonical Wg signal inhibition. Arr, Arrow; Fz2, Frizzled2.

To further investigate how Wg signaling releases the Ras repression mediated by Cno, we examined the possible function of Dsh, which is activated upon Wg signaling, in this process. We found that diP-MAPK expression was lost in *dsh^V26^* lof mutants and expanded in *dsh* gof mutants (Fig. S4). Since Dsh physically interacted with the Ras-binding domain of Cno (Fig. 5, see also Fig. 8A), we next tested whether Dsh competes with Ras for binding Cno. We found that addition of increasing amounts of Dsh led to reduced amounts of Ras^V12^ (Ras^Act^) co-immunoprecipitated with Cno (Fig. 7I). Thus, Dsh was able to compete with Ras for binding Cno. Altogether, these experiments strongly suggested that Wg-Dsh signaling activates *eve* during muscle/heart progenitor specification both by direct binding of dTCF/Arm to the *eve* muscle enhancer [55] and by releasing the Ras repression mediated by Cno.

## Discussion

Elucidating crosstalk mechanisms and regulation is fundamental to the understanding of how cells integrate the complex information that they receive from their environment to generate precise cell responses [1]. In this study, we argue that the PDZ-domain containing protein Cno/AF-6 is a key modulator of Ras-MAPK, N and Wg/Wnt signaling pathways cross-communication: (1) Cno is expressed during muscle progenitor formation, (2) Cno colocalizes in vivo and genetically interacts with components of these three pathways, which are critical to muscle progenitor specification and (3) Cno represses each of these pathways through physical interactions with Ras^Act^, Dsh and N. We propose that Cno, through those interactions, coordinates and integrates the information required by cells to adopt the progenitor fate.

### Cno represses Ras-MAPK, N and canonical Wg/Wnt signaling pathways

A body of evidence suggests that Cno/AF-6 represses the Ras-MAPK signaling pathway [34], [35], [59], [60]. In this work, we present further data that strongly support the inhibition of Ras-MAPK signaling by Cno in vivo. The repression of Ras-MAPK signaling by Cno does not account, however, for all the phenotypes detected in *cno^2^* mutant embryos, such as cases of Eve^+^ progenitor losses (i.e. if Cno were merely inhibiting Ras, in *cno^2^* mutant embryos there would be more positive Ras signaling and, thus, only more Eve^+^ progenitors should be found). In this work, we provide data that support an inhibitory effect of Cno on N signaling during muscle and heart progenitor specification. Since N inhibits progenitor fate, the cases of Eve^+^ progenitors losses found in *cno* ^2^ mutants can be explained by the increase in N signaling in *cno* ^2^ mutants. Indeed, an increase in *E(spl)-m8* expression, a main effector of N signaling, was detected in *cno* ^2^ mutants (Fig. S2C, S2D). Given that Cno interacted physically with N and that the overexpression of Cno led to an accumulation of N at the membrane, one possibility is that Cno inhibits N signaling by impeding the cleavage of N from the membrane. Other possibilities are that Cno interferes with N endocytosis and vesicular trafficking, both key processes in N signaling regulation [61], [62]. Further experiments are required to test these hypotheses.

The repression of both Ras-MAPK and N signaling by Cno still cannot account for all the phenotypes observed in *cno^2^* mutants, such as the extra Eve^+^ cells that appeared in intersegmental regions. This phenotype is characteristic of Wg overexpression suggesting that, in wildtype conditions, Cno is also repressing Wg signaling. Indeed, we present data in this work that support a Cno inhibitory effect on canonical Wg signaling. Since Dsh and Cno interacted physically, one possibility is that Cno interrupts Wg signaling at the level of Dsh, perhaps by sequestering Dsh away from other pathway components or by diverting it to other non-canonical roles.

Hence, a repressive effect of Cno on both negative (N) and positive (Wg and Ras-MAPK) signals required for progenitor fate could ultimately explain the variable phenotypes found in *cno* lof and gof mutants (Fig. 2). For example, in *cno^2^* mutants there will be more positive Ras and Wg signals, but also more negative N signal (Fig. 3K); and Cno overexpression will lead to less positive Ras and Wg signals, but also to less repressive N signal. Thus, the final net balance of positive and negative signals would determine the variable phenotype observed in *cno^2^* zygotic null mutant embryos. A maternal contribution of *cno* has been reported [63]. This maternal contribution might be masking the complete lof phenotype of *cno* (i.e. a “not-variable” phenotype). However, we favour the hypothesis that the variable phenotype observed in *cno* zygotic mutants is due to the Cno regulation of both positive and negative signals required for progenitor fate. Thus, we would expect that different thresholds of Cno protein (from the complete lof condition to a gof condition) would cause a variable phenotype. Indeed both a hypomorph allele of *cno* (*cno^mis1^*) and *cno* gof showed that variable phenotype (Fig. 2G). Given the reported relationships between Cno and Ras, N or Wg in different developmental contexts, Cno could be a general link and modulator of these signaling pathways throughout development.

### How is Cno linking Ras, N and Wg signaling pathways?

We propose a working model to explain how Cno is mediating Ras, N and Wg cross-talk during progenitor specification (Fig. 8B). First, just before Wg signaling begins, Cno directly interacts with Ras^Act^ and inhibits its ability to signal. As a result of Wg signal activation, L'sc starts to be expressed in the mesoderm [10]. Then, L'sc upregulates Dl expression in the mesoderm (A.C., unpublished data, not shown in the model), which in turn activates N in nearby cells, initiating the process of lateral inhibition in the equivalence groups. At the same time, Dsh activation by Wg would result in a competition between Dsh and Ras for the Cno Ras-binding domain. Consequently, Ras repression by Cno is released; this leads to the upregulation of L'sc and to the activation of the identity gene *eve*, which requires both Ras-MAPK and Wg signaling for its expression. The binding of Cno to the Dsh DEP domain facilitates the localization of both Dsh and Cno at the membrane. This would have two consequences: (1) the inactivation of the Wg signaling through the canonical pathway and (2) the repression of N signaling by Cno physical interaction. Whether Dsh is phosphorylated or unphosphorylated when forming part of the Cno-Dsh complex is something that will be pursued in future studies.

Thus, Cno links Ras and N pathways through its binding to Dsh: Dsh-Cno interaction would facilitate both Ras signaling activation and N signaling inhibition. By directly interacting with Ras^Act^, Dsh and N and by locating these proteins in close proximity, Cno would actively coordinate Wg with Ras and N signaling pathways, allowing their cross-communication and modulating their relative signal thresholds throughout progenitor specification. Our data imply that Wg/Dsh is not only a critical permissive signal for the initial mesoderm patterning, but also this signal would play an important role later, during the process of lateral inhibition, in combination with Ras and N. The complexes Cno-Ras, Cno-Dsh, and Cno-N would be in a dynamic equilibrium. The thresholds and relative affinities of all these proteins, their spatio-temporal control and, ultimately, the specific cell context would dictate the binding state and activity of Cno.

### Cno role as a mediator between Wg-Dsh and N signaling pathways

Dsh has been shown to bind N by using yeast two-hybrid assays [49], [50]. Dsh has also been shown to coimmunoprecipitate with N in vivo [51]. However, both our yeast two-hybrid and in vitro co-IP experiments did not support a direct Dsh-N interaction. Our experiments showed that Cno physically interacts with N by using both yeast two-hybrid and co-IP assays. Thus, the described inhibitory effect of Dsh on N signaling [49] is likely to be mediated through Cno, by directly binding N. Yet, other possibilities could explain our observations. For example, a weak physical interaction between Dsh and N may require the presence of Cno to strengthen it; this could be achieved by the simultaneous binding of Cno to Dsh and N. Another possibility is that the complex Cno-Dsh facilitates a post-translational modification of Dsh required for binding N, which is not occurring under our experimental conditions. Indeed, Dsh is a phosphoprotein that becomes hyperphosphorylated upon Wg signaling [64]. Our working model takes into account the following observations: Cno bound both Dsh and N, Dsh did not clearly bind N, both Dsh and Cno interacted antagonistically with N and the interaction between Dsh and Cno was important for N inhibition. Additional experiments are required to further clarify the functional relationships among these proteins.

### Conclusions

Multiple points of cross-communication among signaling pathways are crucial to ensure a fine-tuned regulation of signaling networks. Integration of signals at the enhancer level has been shown to be an essential mechanism to achieve specific and coordinated responses [55]. In this study we show how regulation at the membrane level through Cno and Dsh, two PDZ domain-containing proteins, is also critical to elicit signaling pathways integration. Multiprotein complex formation around PDZ-based scaffolds at specific sub-membrane locations is known to be decisive for signal transduction rate and fidelity [29], [30]. Thus, PDZ proteins are excellent candidates as points of crosstalk at the membrane level. Here, we show a dynamic role for a PDZ protein, modulating the thresholds of multiple signal transduction pathways in vivo. Future investigation will allow a further understanding of how these proteins organize and regulate the complex cell networks of signaling pathways.

## Materials and Methods

### Drosophila strains and genetics

The following mutant stocks were used: *cno^2^*, *cno^mis1^* [24], *Df(3L)^6-7^*, *Dl^X^*, *aos^*Δ*7^*, *pnt^*Δ*88^*, *wg^CX4^*, *dsh^V26^*, *Ras1^e2F^,* *N^X81K1^*. Ectopic expression was achieved with the GAL4-UAS system [65] and the following fly lines: *twi*-GAL4, *Dmef2*-GAL4, *UAS*-*Ras1^Ac^* ^t^, *UAS*-*N^Act^*, *UAS*-*wg*, *UAS*-*cno* [35], *UAS-cno*Δ*N* [53], *UAS*-*arm^S10^, UAS*-*dsh* and *UAS*-*dsh*Δ*DEP* [52]. Two *UAS-cno* transgenes were used in combination to analyze the effect on Dsh expression. All the crosses Gal4-UAS were carried out at 29°C. *twi*-GAL4 and *Dmef2*-GAL4 drivers were used in combination for all overexpression experiments, except for overexpression of Ras1 in a *cno^2^* homozygous background; in this case, only the *twi*-GAL4 driver was employed. Other lines used in this work were: *twi-CD2* [66] and *dsh-GFP* [52]. *yw* was used as the reference wildtype strain. Balancer chromosomes containing different *lacZ* transgenes were used for identification of homozygous mutant embryos.

### Immunohistochemistry, in situ hybridization and microscopy

Embryo fixation, antibody staining and in situ hybridization were carried out by standard protocols [10]. The following primary antibodies were used: rabbit *α*-Cno, 1/500 [25]; rat *α*-Cno [53], mouse *α*-CD2, 1/800 (Serotec); rat *α*-L'sc, 1/800 [67]; rabbit *α*-Eve, 1/3000 [68]; mouse *α*-Eve, 1/25 (Hybridoma Bank); guinea pig *α*-Eve, 1/200 [69]; guinea pig *α*-Kr, 1/2000 [69]; mouse *α*-Dl, 1/40 (M. Muskavitch); rabbit *α*-Htl 1/1000 [42]; rabbit *α*-β-galactosidase 1/100000 (Cappel); mouse *α*-β-galactosidase 1/8000 (Promega); mouse *α*-diP-MAPK, 1/400 (Sigma); rabbit *α*-Twi, 1/1000 (S. Roth); mouse *α*-N 1/100 (Hybridoma Bank); mouse *α*-GFP, 1/400 (Clontech); rat *α*-Dsh, 1/500 [70], mAb 3E2 [5]. For immunostaining with the *α*-Cno antibody, embryos were fixed by using the heat-methanol method [71]. L'sc and diP-MAPK signals were enhanced by use of Tyramide Signal Amplification reagents (New England Nuclear). Fluorescent images were recorded by using a Zeiss LSM 510 Axiovert 100M microscope and assembled by using Adobe Photoshop. Blinded scoring of Notch and Dsh staining in the wildtype and *cno* overexpression studies (Fig. 6) was performed.

### Statistical analysis

The phenotypic effects of individual mutations in various genetic backgrounds were tested for significance (P<0.05) with pairwise comparisons of genotypes by a standard Fisher test.

### Yeast two-hybrid assays and cloning

Yeast transformations and *β*-galactosidase quantitative liquid assays were carried out following Clontech User Manual protocols. Dsh constructs (DSH-FLN and DSH-DEP, in the bait vector pEG202/LexA) as well as N constructs (ICN1 and ICN2, in the prey vector PJG-4/pB42AD) were previously described [49]. DSH-DIZ comprising amino acids (aa) 1 to 393 was subcloned in the same bait vector, LexA. Cno-NH, in the prey vector pACT2, and Ras^V12^, in the bait vector pAS2-1, were previously described [35]. Other Cno constructs used in this work were: Cno-RA (containing the two Ras-associating domains, aa 1-355), Cno-MP (comprising Myo-like and PDZ domains, aa 503-935), Cno-PDZ (aa 836-935), Cno-M (aa 503-754) and Cno-*MP (comprising a truncated Myo-like domain and the whole PDZ domain, aa 521-935). All these Cno fragments were PCR amplified, subcloned into the prey vector pACT2 and verified by sequence analysis. Western Blots confirmed expression of the fusion proteins (not shown). Cno NH, Cno-RA and Cno-MP were also subcloned in the bait vector LexA. Yeast strains L40 [72] or PJ69-4A [73] were transformed with the corresponding bait vector. Four to six colonies were tested for basal *β*-galactosidase activity and one picked for further transformation with the prey vector (with the insert and without any insert as control). Six colonies, harboring bait and prey vectors, were assayed for *β*-galactosidase activity. This assay was repeated at least four times with independent protein extracts. The activity was represented as fold activation with respect to the empty vector.

### Coimmunoprecipitation assays

Co-IP's from embryonic lysates: Proteins extracts from 0–7 h-old fly embryos were homogenized and incubated for 45 min at 4°C in lysis buffer (50 mM Tris pH 8, 150 mM NaCl, 1 mM EDTA, 1% Triton X-100, 0.1% SDS) containing phosphatase and protease inhibitors (1 mM NaF, 100 mM NaVO_4_, 1 mM PMSF and protease inhibitor complete cocktail from Roche). Protein extracts were incubated overnight at 4°C with the appropriate antibody (Rat *α*-Dsh or Rb *α*-Notch) 1/1000. Extracts were filtered and incubated with Protein A beads (Sigma) 2 h at room temperature (RT). Beads were washed 2 times (15 min each) and bound proteins were separated on SDS gels and immunoblotted. Rat (or Rb) *α*-Cno antibody and HRP-coupled secondary antibodies were used 1/5000 and 1/2000, respectively. Co-IP's with in vitro translated proteins: Proteins were translated using the TNT Coupled Reticulocyte Lysate Systems (Promega) according to the manufacturer's instructions, in presence or absence of L-[^35^S]-Methionine (Amersham). The in vitro coimmunoprecipitations were carried out following the directions of BD Biosciences Matchmaker Co-IP Kit User Manual. A N construct containing the whole intracellular domain was used for the co-IP's (Notch-“intra”; Fig. 5C). Proteins were Myc (Cno and N) or HA tagged (Dsh, Ras^V12^ and Cno for the co-IP experiment with N-Myc). *α*-HA and *α*-Myc antibodies were from BD Biosciences. For the competition assay, in vitro translated proteins Cno and ^35^S-Methionine-labeled Ras^V12^ were incubated at RT for one hour. Then, different amounts of in vitro translated Dsh were added. After one hour at RT, 10 ml of c-Myc Monoclonal Antibody was added; the reactions were incubated overnight at 4°C for co-IP Cno-Dsh/Cno-Ras^V12^ complexes. These complexes were resolved on 10% SDS-PAGE minigels. The amount of ^35^S-Methionine-labeled Ras^V12^ coimmunoprecipitated with Cno was detected by autoradiography (Kodak BioMax MR).

## Supporting Information

Figure S1 The expression of different proteins required for progenitor specification is both upregulated and downregulated in cno2 zygotic null mutants. (A–R) Panels show 63× magnifications of three hemisegments, in a lateral view, of stage 11 embryos. (A) L'sc (green) and Eve (red) colocalize in a dorsal progenitor singled out in three adjacent hemisegments. In wildtype embryos, only L'sc is expressed in the preC2 (inset; see also Fig. 1). (E) In cno2 mutants, L'sc is maintained in the extra progenitors specified and Eve is detected along with L'sc in the preC2 (inset). (B, F) L'sc expression in dorsal progenitors (arrows) also fails in some cno2 mutants (arrowheads in F) (NS, nervous system). (C) Kr (green) and Eve (red) colocalize in a dorsal muscle founder cell per hemisegment (yellow cells); (G) in cno2 mutants extra Kr+/Eve+ expressing cells are detected (arrows). (D, H) A fraction of cno2 mutants also shows loss of Kr+-expressing founder cells (arrowhead in H). (I–R) Dl (green in I, J, M–P) and Htl (green in K, L, Q, R) expression are both upregulated (arrows in O, R) and downregulated (arrowheads in P, R) in cno2 mutants. (I, J, K, L) Dl and Htl wildtype expression. Eve+ dorsal progenitors are shown (red) as reference. (S, T) 40× magnifications, in lateral views, of late stage 14 embryos showing Eve expression. (S) In wildtype embryos, Eve is detected in two pericardial cells (arrows) and one dorsal muscle (dotted line) per hemisegment. (T) In cno2 mutant embryos both loss (arrowheads) and muscle duplications (dotted lines) are detected; the number of Eve+ pericardial cells is also affected (arrows). (U, V) 20× (U) and 40× (V) magnifications of a stage 17 cno2 mutant embryo, in a lateral view, stained with mAb 3E2 (stains all muscles). In wildtype hemisegments (asterisk), four lateral transverse (LT) muscles are detected. In some hemisegments LT muscles are lost (arrowhead) and in other hemisegments LT muscles are duplicated (arrows).
(5.29 MB PDF)

Figure S2 The N effector E(spl)-m8 is upregulated in dshV26 null mutants and in cno2 null mutant embryos. (A, B) The most dorsal mesoderm of three hemisegments of stage 11 embryos is shown. (A) In situ hybridization analysis reveals expression of E(spl)-m8 in the embryonic mesoderm. (B) In dshV26 null mutants (germ line clones), E(spl)-m8 is widely expressed in the mesoderm. (C, D) The most dorsal mesoderm of four hemisegments of stage 11 embryos is shown. Eve protein appears in brown and E(spl)-m8 mRNA in blue. (C) In wildtype (WT) embryos E(spl)-m8 is detected in Eve+ cells (arrows). (D) In cno2 null mutants, E(spl)-m8 is expressed in more cells (arrows). In one hemisegment (arrowhead), the increase in E(spl)-m8 correlates with the loss of Eve expression. as, amnioserosa; tp, tracheal pits.
(7.11 MB PDF)

Figure S3 Cno represses Ras-MAPK signaling pathway. Three hemisegments (lateral views) of stage 11 embryos are shown at high magnification (63×). (A–D) In wildtype embryos, diP-MAPK expression (green) is restricted along with Eve (blue) to the progenitor. (E–L) An expansion of diP-MAPK expression is detected both in cno2, aos*Δ*7 (E–H) and in cno2, DlX (I–L) transheterozygotes (arrows). The transcription factor Twist (Twi, red) marks mesoderm. tp, tracheal pits.
(3.37 MB PDF)

Figure S4 Dsh is required for MAPK activity. A 63× magnification of the dorsal mesoderm of three hemisegments is shown. Eve appears in red, diP-MAPK in green and colocalization of both in yellow. (A) In dshV26 null mutants, diP-MAPK expression is not detected in the dorsal mesoderm. Asterisks (*) mark the normal position of Eve+/diP-MAPK+ cells. (B) Dsh*Δ*DEP overexpression in the mesoderm leads to expansion of diP-MAPK (arrows). Dsh*Δ*DEP contains the region that bound the Ras-associating domain of Cno. tp, tracheal pits. (Wildtype diP-MAPK/Eve expression is shown in Fig. S3).
(2.76 MB PDF)

Figure S5 Two independently generated Cno anti-sera coimmunoprecipitate Cno protein with Dsh (double arrow). Both Cno anti-sera recognize three specific bands (arrows in the “Input line”). The Rabbit α-Cno anti-serum recognizes an additional, non specific band, which is not detected by the Rat α-Cno anti-serum. The presence of multiple bands for Cno in the input line has been already published (Takahashi et al., 1998, Mech. Dev. 78, 97–111; see Figure 11 A).
(0.60 MB PDF)
