# Supplementary figures and images for: The PDZ Protein Canoe/AF-6 Links Ras-MAPK, Notch and Wingless/Wnt Signaling Pathways by Directly Interacting with Ras, Notch and Dishevelled
Source: PLoS One. 2006 Dec 20;1(1):e66. doi: 10.1371/journal.pone.0000066 (PMC1762375; doi:10.1371/journal.pone.0000066)

# Supporting Figure 1

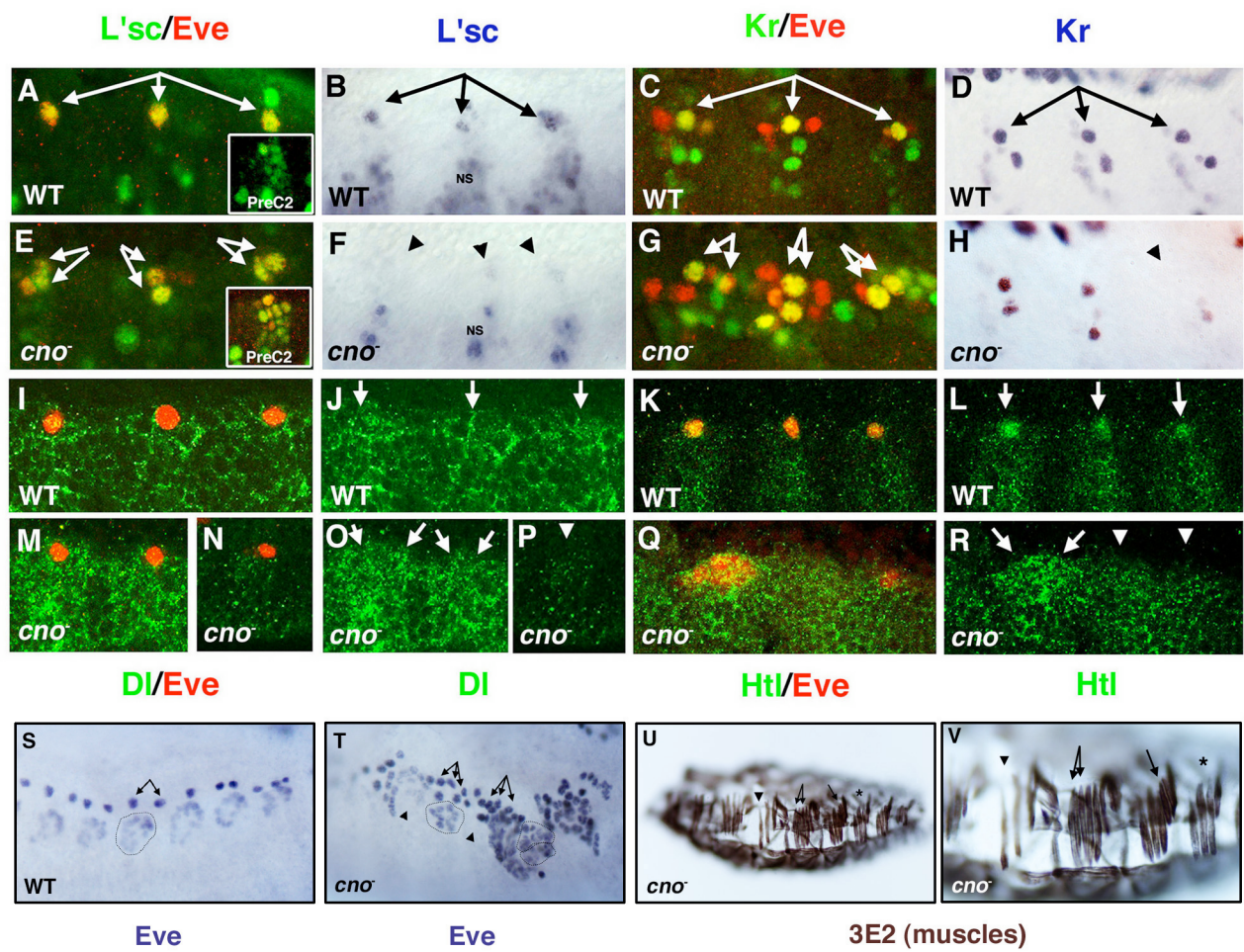

Supplement: Figure S1 — The expression of different proteins required for progenitor specification is both upregulated and downregulated in cno2 zygotic null mutants. (A–R) Panels show 63× magnifications of three hemisegments, in a lateral view, of stage 11 embryos. (A) L'sc (green) and Eve (red) colocalize in a dorsal progenitor singled out in three adjacent hemisegments. In wildtype embryos, only L'sc is expressed in the preC2 (inset; see also Fig. 1). (E) In cno2 mutants, L'sc is maintained in the extra progenitors specified and Eve is detected along with L'sc in the preC2 (inset). (B, F) L'sc expression in dorsal progenitors (arrows) also fails in some cno2 mutants (arrowheads in F) (NS, nervous system). (C) Kr (green) and Eve (red) colocalize in a dorsal muscle founder cell per hemisegment (yellow cells); (G) in cno2 mutants extra Kr+/Eve+ expressing cells are detected (arrows). (D, H) A fraction of cno2 mutants also shows loss of Kr+-expressing founder cells (arrowhead in H). (I–R) Dl (green in I, J, M–P) and Htl (green in K, L, Q, R) expression are both upregulated (arrows in O, R) and downregulated (arrowheads in P, R) in cno2 mutants. (I, J, K, L) Dl and Htl wildtype expression. Eve+ dorsal progenitors are shown (red) as reference. (S, T) 40× magnifications, in lateral views, of late stage 14 embryos showing Eve expression. (S) In wildtype embryos, Eve is detected in two pericardial cells (arrows) and one dorsal muscle (dotted line) per hemisegment. (T) In cno2 mutant embryos both loss (arrowheads) and muscle duplications (dotted lines) are detected; the number of Eve+ pericardial cells is also affected (arrows). (U, V) 20× (U) and 40× (V) magnifications of a stage 17 cno2 mutant embryo, in a lateral view, stained with mAb 3E2 (stains all muscles). In wildtype hemisegments (asterisk), four lateral transverse (LT) muscles are detected. In some hemisegments LT muscles are lost (arrowhead) and in other hemisegments LT muscles are duplicated (arrows). (5.29 MB PDF) [file pone.0000066.s001.pdf]

## Supporting Figure 2

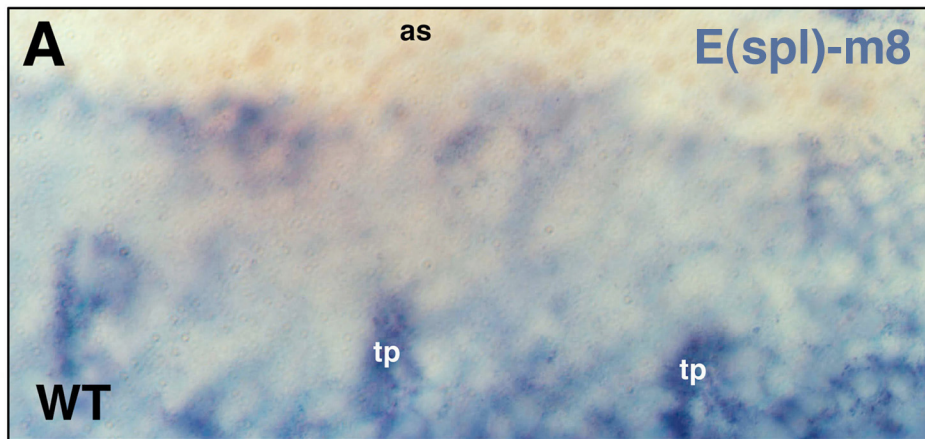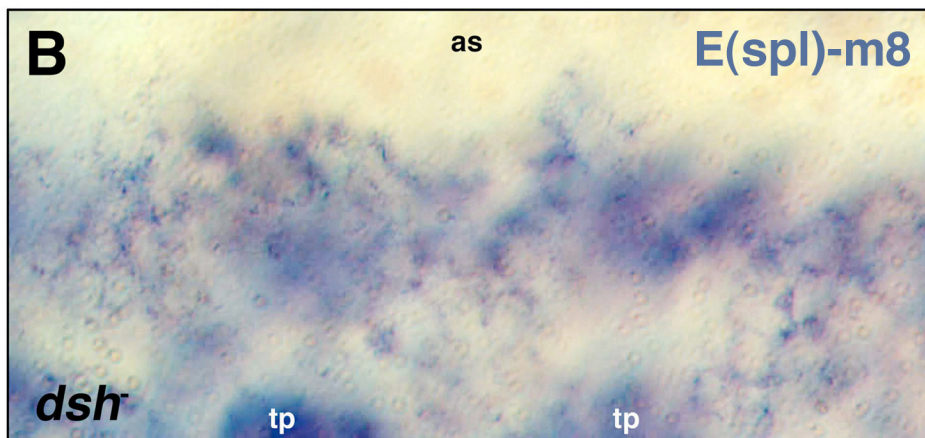

### **E(spl)-m8/Eve**

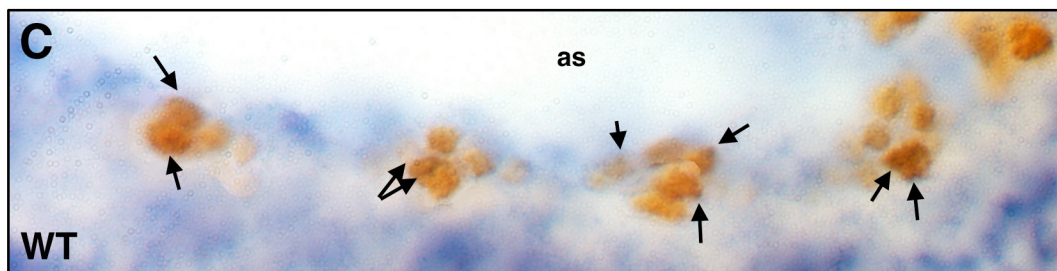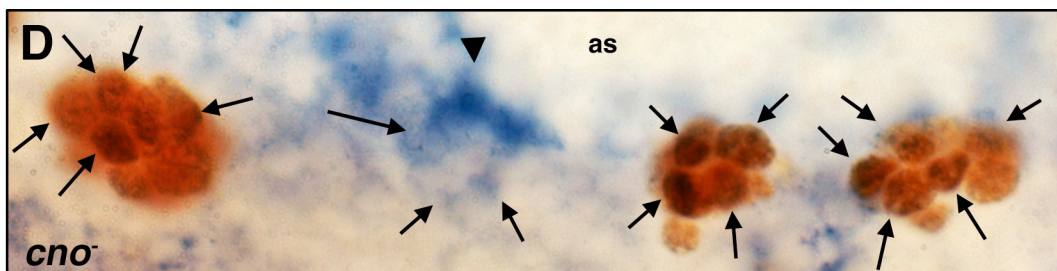

Supplement: Figure S2 — The N effector E(spl)-m8 is upregulated in dshV26 null mutants and in cno2 null mutant embryos. (A, B) The most dorsal mesoderm of three hemisegments of stage 11 embryos is shown. (A) In situ hybridization analysis reveals expression of E(spl)-m8 in the embryonic mesoderm. (B) In dshV26 null mutants (germ line clones), E(spl)-m8 is widely expressed in the mesoderm. (C, D) The most dorsal mesoderm of four hemisegments of stage 11 embryos is shown. Eve protein appears in brown and E(spl)-m8 mRNA in blue. (C) In wildtype (WT) embryos E(spl)-m8 is detected in Eve+ cells (arrows). (D) In cno2 null mutants, E(spl)-m8 is expressed in more cells (arrows). In one hemisegment (arrowhead), the increase in E(spl)-m8 correlates with the loss of Eve expression. as, amnioserosa; tp, tracheal pits. (7.11 MB PDF) [file pone.0000066.s002.pdf]

## Supporting Figure 3

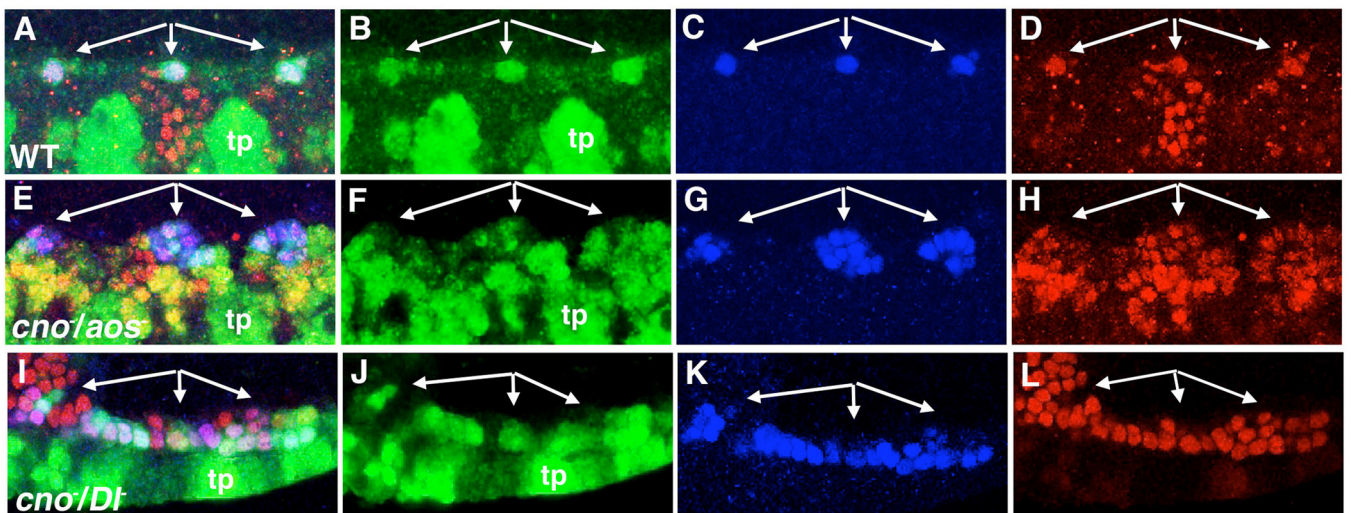

Supplement: Figure S3 — Cno represses Ras-MAPK signaling pathway. Three hemisegments (lateral views) of stage 11 embryos are shown at high magnification (63×). (A–D) In wildtype embryos, diP-MAPK expression (green) is restricted along with Eve (blue) to the progenitor. (E–L) An expansion of diP-MAPK expression is detected both in cno2, aosΔ7 (E–H) and in cno2, DlX (I–L) transheterozygotes (arrows). The transcription factor Twist (Twi, red) marks mesoderm. tp, tracheal pits. (3.37 MB PDF) [file pone.0000066.s003.pdf]

## Supporting Figure 4

diP-MAPK/Eve

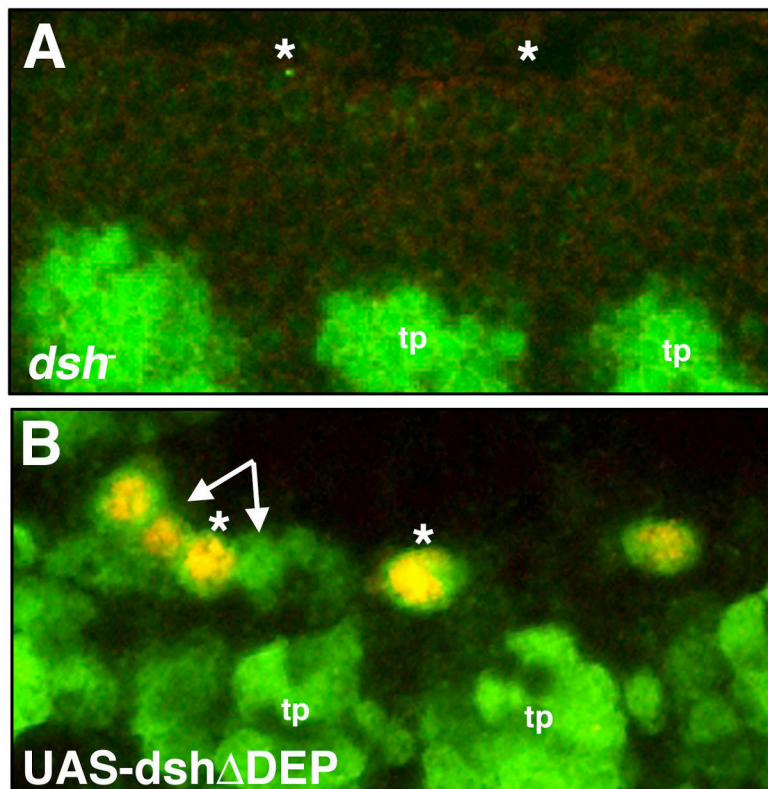

Supplement: Figure S4 — Dsh is required for MAPK activity. A 63× magnification of the dorsal mesoderm of three hemisegments is shown. Eve appears in red, diP-MAPK in green and colocalization of both in yellow. (A) In dshV26 null mutants, diP-MAPK expression is not detected in the dorsal mesoderm. Asterisks (*) mark the normal position of Eve+/diP-MAPK+ cells. (B) DshΔDEP overexpression in the mesoderm leads to expansion of diP-MAPK (arrows). DshΔDEP contains the region that bound the Ras-associating domain of Cno. tp, tracheal pits. (Wildtype diP-MAPK/Eve expression is shown in Fig. S3). (2.76 MB PDF) [file pone.0000066.s004.pdf]

## Supporting Figure 5

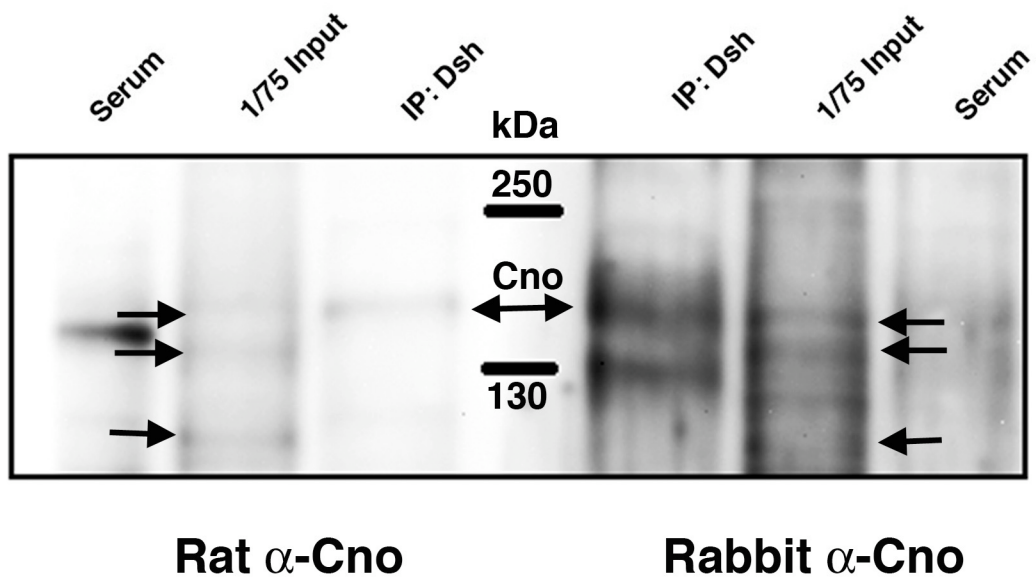

Supplement: Figure S5 — Two independently generated Cno anti-sera coimmunoprecipitate Cno protein with Dsh (double arrow). Both Cno anti-sera recognize three specific bands (arrows in the “Input line”). The Rabbit α-Cno anti-serum recognizes an additional, non specific band, which is not detected by the Rat α-Cno anti-serum. The presence of multiple bands for Cno in the input line has been already published (Takahashi et al., 1998, Mech. Dev. 78, 97–111; see Figure 11 A). (0.60 MB PDF) [file pone.0000066.s005.pdf]
